# Psychobiotics and the Microbiota–Gut–Brain Axis: Where Do We Go from Here?

**DOI:** 10.3390/microorganisms12040634

**Published:** 2024-03-22

**Authors:** Sylvie Binda, Annie Tremblay, Umar Haris Iqbal, Ola Kassem, Mélanie Le Barz, Vincent Thomas, Stéphane Bronner, Tara Perrot, Nafissa Ismail, J.Alex Parker

**Affiliations:** 1 Lallemand Health Solutions, 19 Rue des Briquetiers, BP 59, 31702 Blagnac, France; mlebarz@lallemand.com (M.L.B.); vthomas@lallemand.com (V.T.); 2 Rosell Institute for Microbiome and Probiotics, Lallemand Health Solutions, 6100 Royalmount Avenue, Montreal, QC H4P 2R2, Canada; atremblay@lallemand.com (A.T.); iumarharis@lallemand.com (U.H.I.); okassem@lallemand.com (O.K.); sbronner@lallemand.com (S.B.); 3 Department of Psychology and Neuroscience, Dalhousie University, Halifax, NS B3H 4R2, Canada; tara.perrot@dal.ca; 4 Department of Psychology, University of Ottawa, Ottawa, ON K1N 6N5, Canada; nafissa.ismail@uottawa.ca; 5 Département de Neurosciences, Université de Montréal, Montreal, QC H3T 1J4, Canada; ja.parker@umontreal.ca

**Keywords:** psychobiotics, microbiota–gut–brain axis, stress, early-life stress, neuropsychiatric disorders, neuroinflammation, microglia, metabolic syndrome, obesity

## Abstract

The bidirectional relationship between the gut microbiota and the nervous system is known as the microbiota–gut–brain axis (MGBA). The MGBA controls the complex interactions between the brain, the enteric nervous system, the gut-associated immune system, and the enteric neuroendocrine systems, regulating key physiological functions such as the immune response, sleep, emotions and mood, food intake, and intestinal functions. Psychobiotics are considered tools with the potential to modulate the MGBA through preventive, adjunctive, or curative approaches, but their specific mechanisms of action on many aspects of health are yet to be characterized. This narrative review and perspectives article highlights the key paradigms needing attention as the scope of potential probiotics applications in human health increases, with a growing body of evidence supporting their systemic beneficial effects. However, there are many limitations to overcome before establishing the extent to which we can incorporate probiotics in the management of neuropsychiatric disorders. Although this article uses the term probiotics in a general manner, it remains important to study probiotics at the strain level in most cases.

## 1. Introduction

The gut microbiota is composed of a highly complex community of microorganisms residing in the gastrointestinal (GI) tract of humans and other animals. Most of the microbiota is found in the large intestine, with a smaller fraction residing in the stomach and small intestine. The lifelong symbiotic relationship between microorganisms and the host begins as early as the time of birth, perhaps even in utero [1]. While the host provides the habitat and nutrition, these microorganisms return the favor with various significant benefits. The GI benefits provided by the resident microbiota include supporting digestion and metabolism, vitamin synthesis, maintaining the epithelial integrity of tight junctions (thereby preventing the absorption of harmful molecules or pathogens), colonizing the mucosal layer and competing with pathogens for food and space, and supporting the development of immunity. The systemic benefits of probiotics include enhancing the immune system and, for psychobiotics, influencing gut–brain communication to regulate mood, cognitive and neurological functions, and even brain structures [2,3,4].

**Figure 1:**
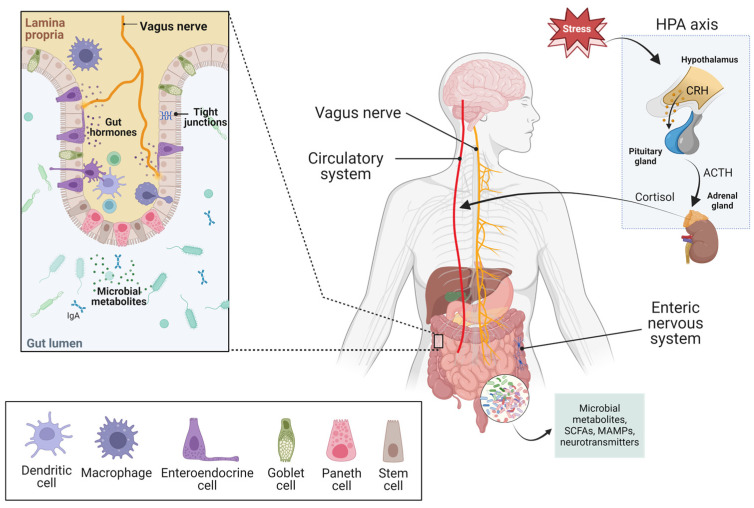
Schematic representation of the main components of the MGBA. The microbiota acts in the gut lumen and on the epithelial mucosa via the secretion of a variety of metabolites, including, but not restricted to, SCFAs and neurotransmitters. The microbial metabolites can cross the epithelial barrier to reach the lamina propria and the circulation. Other metabolites act directly on the epithelial barrier to strengthen tight junctions and stimulate the production of neuroendocrine and immune mediators that will influence vagal afferents or reach the circulation. In the lamina propria, immune cells secrete anti-inflammatory cytokines in response to the specific microbial signals received by the dendritic cells. Stress activates the HPA axis, which controls the circulating concentrations of cortisol, and affects intestinal motility through communication with the enteric nervous system.

Psychobiotics are defined as probiotics that confer mental health benefits to the host when consumed in a particular quantity through the interaction with commensal gut bacteria. Over the last decade, interest in psychobiotics has significantly increased, leading to major advances in understanding their therapeutic potential in indications related to the MGBA. This bidirectional communication that exists between the brain and gut microbiota is thought to be primarily mediated by the enteric nervous system, the hypothalamic–pituitary–adrenal (HPA) axis, and the central and peripheric nervous systems, with influences from immune, endocrine, and molecular pathways (Figure 1) [5,6,7]. Numerous studies have associated the administration of psychobiotics with positive effects on areas of stress, anxiety, neuroinflammation, neurodegenerative diseases such as Alzheimer’s and Parkinson’s diseases (AD and PD), in addition to GI diseases [8,9,10,11,12]. The mechanism by which psychobiotics confer these benefits has been suggested to be mediated through their regulation of neurotransmitters such as serotonin, gamma-aminobutyric acid (GABA), brain-derived neurotropic factor (BDNF), as well as short-chain fatty acids (SCFAs) and enteroendocrine hormones [13,14,15,16,17]. Psychobiotics have also been shown to impact inflammatory pathways by normalizing the levels of pro-inflammatory cytokines as well as inducing increased amounts of anti-inflammatory cytokines such as IL-10 [14,18]. In addition to their anti-inflammatory role, psychobiotics have been shown to reduce the activation of the HPA axis in response to stressors [19,20,21]. 

It is well recognized that through the concept of interoception, the brain can sense and process information related to the internal physiological state of the body [22,23]. This was previously thought to be primarily mediated by fine, unmyelinated vagal and sympathetic afferent neurons. However, we now know that besides those direct neurons, the gut microbes and their metabolites provide a key source of such interoceptive information; psychobiotics can affect the brain through vagal afferents since some of their effects can be alleviated by vagotomy in animal models [24]. Interestingly, in addition to perception, the gut microbiota can even influence the anatomical structure and development of the brain, which subsequently impacts physiological functions, as shown in animal models of early-life stress (ELS). Abnormalities in delicately tuned interoceptive signaling could result in disordered MGBA communications and disease conditions such as irritable bowel syndrome (IBS), functional dyspepsia, chronic abdominal pain, psychiatric disorders, and neurodegenerative (NDDs) and developmental disorders [6,25].

A solution, as proposed also for GI diseases, would be to restore the “normal” or baseline gut microbiota composition and functions, and/or to restore proper communication between the brain and the gut by correcting the imbalanced microbiome population (aka dysbiosis) to that observed in healthy individuals. Accordingly, psychobiotics have emerged as potential tools to mitigate the symptoms of various mental and neuropsychiatric and neurodegenerative conditions. 

## 2. Gut Microbiota and Neurodegenerative Diseases: Crosstalk and Potential Mechanisms

To date, there is still no consensus regarding the definition of a healthy gut microbiota composition given the wide inter-individual variability, even in people considered healthy. However, experts agree that some bacterial genera or species are generally recognized as beneficial, such as *Faecalibacterium prausnitzii*, while other are usually associated with pathological or proinflammatory conditions (pathobionts). Additionally, a strong homogeneity is now well-recognized in the overall activities of the gut microbiota of healthy individuals. Thus, taxonomic diversity is much greater than functional diversity. This redundancy of functions between different species ensures the maintenance of essential activities. 

During the past decades, the pivotal role of the gut microbiota homeostasis and functions has been demonstrated in a plethora of health axes, from the traditional GI system itself to metabolic health, overall immunity and more recently to mental health [26] to only cite a few. In fact, the bidirectional link between the gut microbiota and the functioning of the central nervous system (CNS) was highlighted at the beginning of the 2000s in studies conducted in gnotobiotic mice models [26,27]. Since this pioneering work, numerous preclinical, clinical and epidemiological studies have highlighted several mechanisms involving neural signaling pathways (enteric and CNS), the endocrine system and immunity (detailed later in this review), allowing a certain homeostasis in the MGBA regulation.

In pathophysiological conditions, including the development of NDDs and NPDs, the analysis of the gut microbiota composition had revealed a decrease of its diversity, an altered profile in favor of deleterious genera and species, usually associated with the release of proinflammatory or neuroactive microbial metabolites and an increase of gut permeability [28]. In response to environmental or endogenous factors, but also pathobiont colonization, the gut microbiota produces a large variety of bioactive molecules such as SCFAs, long-chain fatty acids, neurotransmitters, microbial toxins, and microvesicles [29]. In a recent clinical trial conducted in India comparing the gut microbiota composition of PD patients to that of their wife, the authors reported a decrease in *Faecalibacterium*, *Blautia* and *Fusicatenibacter* genera, among others [30]. The significant decrease of *Faecalibacterium* relative abundance has been also highlighted in individuals with subjective cognitive decline and the authors concluded that an altered gut microbiota composition may serve as a potential peripheral biomarker of AD’s onset [31,32]. It is consistent with previous trials conducted in individuals suffering from NDDs revealing a lower abundance of SCFA-producing bacteria, including *Prevotella*, *Faecalibacterium*, *Blautia* and *Roseburia* compared to the gut microbiota of healthy controls [33,34,35,36,37]. In the SOD1^G93A^ mouse model of ALS, an increased gut permeability to toxins and a decrease of the relative abundance of the butyrate-producer *Butyrivibrio fibrisolvens* species were reported [38]. Interestingly, the treatment of SOD1^G93A^ mice with butyrate induced a delayed weight loss and increased survival of ALS animals [39]. The effects of SCFAs may be mediated by several mechanisms of action, including their binding to G-protein-coupled receptors (i.e. GPR43, GPR41, GPR109A) and the inhibition of histone deacetylation leading to epigenetic regulations of several gene expressions [40]. Growing evidence revealed that the beneficial effects of SCFAs on blood-brain-barrier (BBB) integrity are also induced through the inhibition of pro-inflammatory pathways involving NF-κB and one potential mechanism is the activation of Nrf2, a redox-sensitive transcription factor (for specific pathway details, please refer to [41]). Regarding acetate, even if it is still controversial, some experts agree that this small molecule is able to cross the BBB, as demonstrated by Frost et al. in vivo [42], and thus acetate may alter the level of the neurotransmitters produced locally, such as glutamate, glutamine and GABA.

Gut microbiota dysbiosis is not only described as a reduction or the absence of beneficial species, but also by the excessive increase of specific commensal species which will induce proinflammatory reactions or even by the invasion of pathobionts. Gut leakiness increase had been reported in NDDs’ patients, as compared with healthy individuals. In PD’s patients, increase in *Enterobacteriaceae*, in particular *Escherichia coli*, has been reported as compared to healthy control, and it was associated with the increase of the expression of genes involved in lipopolysaccharide (LPS) biosynthesis and bacterial secretion [43] (for a review of LPS downstream pathways involved in NDD development, please refer to [44]). Interestingly, it was also associated with an increase of α-Syn in the GI tract [45] and some data suggested that α-Syn may spread via the vagus nerve to the brainstem [46]. Yildirim et al. recently observed that overrepresentation of *Escherichia* was associated with the increase of opportunistic species of *Klebsiella* and *Enterococcus*, as compared with healthy control individuals [47]. Another mechanism related to Gram-negative bacteria was recently reported in the literature. Indeed, new results pointed out a role for small outer membrane vesicles which seem to shuttle bacterial toxins and virulence factor to distant organs, contributing to PD pathogenesis [48]. Similarly, the link between specific pathogens and the onset of NDDs seems to be multifactorial and involves the activation of chronic inflammatory pathways [44], as it was recently highlighted between AD and the presence of *Helicobacter pylori* in the gut, which is no longer only associated with gut disorders but also with mental health [49]. Specific profile of gut microbiota alterations in humans suffering from the different NDDs were gathered in a recent review by Khatoon et al. [50]. 

In addition to LPS, a well-described proinflammatory marker of physiopathologies including NDDs, other gut microbiota metabolites might send signals to the brain through the activation of afferent sensory neurons of the vagus nerve [51]. It is notably the case for by-products of the tryptophan/kynurenin pathway which is directly impacted by gut microbiota metabolism since several bacterial genera can produce and/or metabolize tryptophan. Widner et al. reported that the severity of cognitive function impairment could be directly correlated with kynurenin/tryptophan increase [52] (for detailed information regarding this important pathway, please refer to [51,53]). Trimethylamine-n-oxide (TMAO), derived from the TMA produced by gut bacteria, has been also associated with NDDs. Del Rio et al. revealed the ability of this proinflammatory marker to cross the BBB as it was found in the cerebrospinal fluid of AD patients [54]. 

More recently, Ide et al. carried out a 6-month prospective study in elderly with mild cognitive impairment and demonstrated that periodontitis diagnosis was associated with a marked increase in cognitive decline independently to baseline cognitive state [55]. In fact, LPS from *Porphyromonas gingivalis*, a species commonly found in the oral tract was detected in the brains of AD patients [56]. These results were corroborated by a preclinical trial showing that oral administration of *P. gingivalis* in C57BL/6 mice induced pathological symptoms of AD, as neuroinflammation, neurodegeneration or β-amyloid plaque formation [57]. In addition to that of the gut microbiota, these data raise an important role for the oral microbiota, having the particularity of inducing biofilms, in the onset of NDDs, as recently reviewed [58]. Researchers recently identified new biomarkers in the blood that can be detected up to 10 years before the onset of AD’s symptoms, one of these being GFAP (glial fibrillary acidic protein) [59,60,61] which can be regulated by probiotics intake in animal models [62]. While more research is required to characterize the meaning of this finding, we can speculate that changes in both oral and gut microbiota may be involved earlier than the current timeframe of microbiota composition studies that provide only a snapshot of the existing state without informing on the potential causal role of dysbiosis. 

These potential mechanisms and crosstalk between pathways and systems involved in NDDs are also relevant for other NPDs such as depression and schizophrenia, although the role of the MGBA in most NPDs is still understudied. Microbiota dysbiosis and eubiosis in these conditions also remain to be characterized at the functional level. 

## 3. Psychobiotics and Neuropsychiatric Disorders (NPDs)

A recent systematic review by Ribera et al. (2024) [see [63], and references therein] identified 43 clinical trials assessing the effects of various psychobiotics (probiotics or synbiotics) in clinically diagnosed NPDs. Major depressive disorder (MDD) was the most studied disorder with 17 trials. Other disorders were deemed understudied, which prevented formal conclusions about the benefits of psychobiotics in schizophrenia (10 studies), bipolar disorder (5 studies), anorexia nervosa (4 studies), attention deficit hyperactivity disorder (ADHD) (3 studies), anxiety disorders (2 studies), Tourette syndrome (1 study) and insomnia (1 study). The authors concluded that psychobiotics are beneficial in MDD patients, but that more well-designed studies are required in other indications. Overall, these studies used probiotic formulations containing various amounts of *Lactobacilli* and *Bifidobacteria* strains at various dosages. The psychobiotics strains and formulations that showed a significant effect on depression are listed in Table 1, which includes 9 studies on 8 different psychobiotic strain or formulations. Studies on synbiotics were excluded from this table as prebiotics are not covered herein. Significant improvements are depicted by green background (and underlined), while questionnaires that showed no change are depicted by a red background (not underlined). This table also provides an overview of the formulations and strains that were tested using a variety of questionnaires (assessing depression, anxiety, sleep quality and GI symptoms), highlighting one of the main difficulty inherent to systematic reviews: the differences between studies in terms of assessment tools and psychobiotic regimens do not allow for recommendation of a specific probiotic strain or dose for all NPDs. Typical limitations identified in the systematic review are listed in Box 1.

Box 1 **Typical limitations of psychobiotic studies in NPD indications [26].**

- High variability in strain, dose, and duration of supplementation.
- Use as an adjuvant to pharmacologic treatments or alone, or with nutraceuticals without a corresponding nutraceutical control arm.
- Heterogeneity in the prior or co-administered pharmacologic treatments.
- Heterogeneity in outcome measures and outcome assessment tools.
- Lack of patient-centered outcomes, such as social functioning.

Mechanistic studies in animal models of depression have highlighted the importance of the HPA axis and the vagus nerve in the effect of psychobiotics via the MGBA (Figure 1). In humans, mechanistic data in MDD patients are still scarce. Among studies that included mechanistic outcomes, the common ground was to assess inflammatory markers as well as biochemical markers of glucose metabolism because these pathways, for which many probiotics show regulatory effects, have also been linked with the pathophysiology of several NPDs. For example, in addition to the improvements in depressive symptoms, the blend of *L. acidophilus*, *L. casei* and *B. bifidum* [64] also induced a significant decrease in insulin, HOMA-IR and hs-CRP levels. *Bacillus coagulans* MTCC 5856 intake was associated with a significant reduction in myeloperoxidase levels [65], while a significant reduction in the isoleucine-normalized kynurenine/tryptophan ratio was observed in the Cerebiome® group versus placebo, as well as increased serum BDNF levels and increased appetite without affecting body weight over the 8-week study [66,67,68].

Importantly, Ribera et al. (2024) excluded studies on populations with non-medically diagnosed conditions. However, psychobiotics have been shown to exert positive effects also in sub-clinical contexts [27,28]. In systematic reviews considering both sub-clinical and clinical populations, conclusions are limited by the heterogeneity between populations in addition to the limitations presented in Box 1.

**Table 1:** Questionnaire results for psychobiotic strains and blends that showed significant improvements on depressive symptoms in MDD patients (Adapted from [63]).

Strain/Formulation	Depressive Symptoms		Anxiety Symptoms	Sleep Quality	GI Symptoms	Refs
*L. paracasei* Shirota (8 × 10^10^ CFU)	HDRS	BDI	STAI	PSQI	GSRS	[69]
*B. coagulans* MTCC 5856 (2 × 10^9^ CFUs)	CES-D, CGI, HDRS, MADRS		Not tested	Not tested	IBS-QOL	[65]
*L. plantarum* PS128 (6 × 10^10^ CFUs)	HDRS, DSSS		Not tested	Not tested	Not tested	[70]
*C. butyricum* MIYAIRI 588 © (60 mg)	BDI, HDRS		BAI	Not tested	Not tested	[71]
*L. helveticus* R0052, *B. longum* R0175 (Cerebiome®; 10% and 90% of the composition respectively, 3 × 10^9^ CFUs total)	BDI, MADRS, QIDS-SR16, SHAPS		GAD-7, STAI	PSQI	Not tested	[66,67,68,72]
*S. thermophilus*, *B. breve*, *B. longum*, *B. infantis, L. acidophilus*, *L. plantarum*, *L. paracasei*, *L. delbrueckii* (9 × 10^11^ CFUs total)	HDRS	BDI	STAI	Not tested	GSRS	[73]
*B. breve*, *B. longum*, *P. acidilactici* (4 × 10^9^ CFUs/g; 4 g)	HDRS, MADRS		Not tested	Not tested	GSRS	[74]
*L. acidophilus*, *L. casei*, *B. bifidum* (2 × 10^9^ CFUs each)	BDI		Not tested	Not tested	Not tested	[64]

BAI, Beck’s Anxiety Inventory; BDI, Beck’s Depression Inventory; CES-D, Centre for Epidemiologic Studies Depression scale; CGI, Clinical global impression scale; DSSS, Depression and Somatic Symptoms Scale; GAD-7, General Anxiety Disorder-7; GSRS, Gastrointestinal symptom rating scale; HDRS, Hamilton Depression Rating Scale (also known as the Ham-D); IBS-QOL, Irritable Bowel Syndrome Quality of Life Instrument; MADRS, Montgomery-Asberg Depression Rating Scale; PSQI, Pittsburgh Sleep Quality Index; QIDS-SR16, 16-item Quick Inventory of Depressive Symptomatology-Self-report; SHAPS, Snaith-Hamilton Pleasure Scale; STAI, State-Trait Anxiety Inventory.

One main difference between subclinical and clinical depression is the prescription of antidepressants. These drugs were shown to affect the gut microbiota composition, either beneficially or in a deleterious manner [29]. On one hand, some antidepressants were shown to favor eubiosis and restore microbiota composition, which could be one of their mechanisms of actions. On the other hand, antidepressants, by changing the composition of the gut microbiota, may reduce the treatment efficacy by influencing the absorption, metabolism and activity neuropsychiatric drugs. This could explain the development of treatment resistance in some individuals. Furthermore, the antibacterial activity of antidepressants raises concerns in terms of the development of antibiotic resistance. It would be interesting to figure out which psychobiotics could synergize with specific antidepressants and potentially allow to lower the dose of pharmacological agents while achieving the same therapeutic effect. For instance, several antidepressants are associated with weight gain, which appears to also be mediated by their impact on the microbiota composition by reducing the abundance of taxa involved in weight control [29]. Antidepressants can also modulate the secretome of the gut microbiota, as shown in a study of 290 MDD outpatients treated with citalopram or escitalopram that identified an association between SSRIs and alterations in the metabolic profiles of tryptophan, purine, and tyrosine pathways [30]. Antidepressants can also alter the epithelial mucosal barrier, with some SSRIs associated with an increased or decreased gut permeability and epithelial functions. Considering the importance of the microbiota in the overall response to treatment in patients with depression (a concept that also applies to other neuropsychiatric disorders and other psychotropic drugs), it will be important to further characterize antidepressants-psychobiotics pairings for their efficacy and global effect on the gut microbiota composition.

Overall, despite several encouraging results, the application of psychobiotics in mental and neurological diseases in clinical trials is primarily perceived to be of a supportive nature rather than a treatment. As research progresses towards a better understanding of the holistic nature of mental health maintenance, with reciprocal impacts between stress, sleep, lifestyle factors, eating habits, early-life environment and upbringing conditions, as well as other comorbidities, the integration of psychobiotics to the NPD treatment armamentarium will be facilitated and possibly tailored to the specific modes of actions of each strain or specific mix. For example, Cerebiome® and *L. paracasei* Shirota both improved sleep quality scores on the PSQI. Considering that sleep disruption is well-recognized to contribute to neuropsychiatric disorders’ severity, these psychobiotics should be studied in other conditions or age groups where impaired sleep has been shown to have an impact.

## 4. Psychobiotics and Microbiota in Sleep Quality, Stress and Mental Health

A systematic review by Scott et al. (2021) including 65 studies reported that improving sleep quality leads to better mental health [75] and Staines et al (2022), based on 43 studies, found that improving sleep quality was associated with a reduction of anxiety symptoms [76]. A 2022 systematic review of 34 studies exploring the associations of stress with poor sleep quality and/or insomnia in undergraduate students found a strong association between insomnia and stress, and a moderate pooled association between sleep quality, insomnia and stress [77]. There is not much debate around the negative effects of stress and the importance of good sleep for overall and mental health. However, more research is required to determine the efficacy of specific stress reduction and sleep interventions and assess the effect of incorporating sleep improvement strategies in mental health services [75].

A large evidence base supports the role of psychobiotics against stress-induced GI and behavioral symptoms. It is believed that one of the key factors mediating the adverse effects of stress on mental health is the gut microbiome [78]. In mice, exposure to stressful environmental factors, such as chronic sleep disruption, during puberty induces depression-like behavior. However, probiotic supplementation during puberty significantly mitigated the latter effect in both males and females to a level comparable to rested mice [79,80]. These findings suggest that, as opposed to pharmacologic treatments which have been shown to negatively affect the microbiome, psychobiotics exert their benefits both in the gut and at a systemic level. Exposure to chronic sleep disruption also induces a significant decrease in tryptophan concentration in the prefrontal cortex, and glucose and lactate concentrations in the hippocampus, both of which were mitigated by probiotics supplementation [79]. Probiotics have also been shown to mitigate the detrimental effects of maternal separation in animals, modeling early-life stress and its lifelong consequences [81]. Mental and neurological wellbeing in adulthood is significantly impacted by stress exposure in early life. During this period, ongoing development of the nervous system allows for programming by internal and external events. Based on animal models, it appears that the impact of this programming is not limited to the exposed individual but also imposes a trans-generational effect. For instance, induction of stress during gestation or pregnancy, which subsequently impacts the fetus, can result in epigenetic changes in adult offspring that are then passed on to the subsequent generation [82]. Mechanistic insights from animal studies include altered SCFA production, disruption of T helper 17 cells differentiation and maternal immune activation, or alterations of tryptophan metabolism and serotoninergic signaling [83]. However, which outcomes do we measure, and when, to validate this lifelong and transgenerational process in humans? The study of the role of probiotics on epigenetics in epithelial intestinal cells in vitro suggests that they can modulate the global histone methylation and acetylation status [84], which is of utmost interest from a neurodevelopmental biology perspective.

Nevertheless, for clinical trials, prerequisite research questions have yet to be answered: in what manner do GBA interactions in early life influence the subsequent vulnerability to developmental and neurological disorders, and how do we factor the interindividual differences in resilience abilities in this assessment? Indeed, one hypothesis is that early-life stress would reduce the ability to cope with subsequent life stressors. Moreover, a statistical report published by the U.S. National Institute of Mental Health highlights the age- and sex-dependent nature of mental illnesses. In 2021, just after the devastating worldwide pandemic, the prevalence of mental illness was reported to be higher among females (27.2%) compared to males (18.1%), with young adults aged 18–25 years having the highest prevalence (33.7%). An estimated 49.5% of adolescents had a mental disorder, out of which 22.2% experienced severe impairment and/or distress [85]. In Canada, this age group reported the most important decline in mental health after the pandemic, and those already experiencing poor mental health before COVID-19 were impacted even more [86]. Conceivably, a stressful environment during the early years of life, followed by the physiological changes and psychosocial stressors during puberty, such as significant hormonal changes, heightened emotional sensitivity, academic pressure, social and peer pressure, and concerns about self-image all contributed to the high prevalence of mental illnesses in adolescents and young adults. A main issue pertaining to this group is limited proper diagnosis due to significant physical, emotional, and social changes that make it difficult for caregivers to distinguish potential mental illness symptoms and (ab)normal expected adolescent behaviors. However, in infants and children, the microbiota composition in early life was associated with temperament based on 6 studies, which is important considering that childhood temperament is believed to lay grounds for later personality, behavior and risk of psychopathology [87]. 

A recent systematic review by Augusti et al. (2023) identified 13 longitudinal, cross-sectional and case-control studies assessing the relationship between ELS, either prenatal (4 studies) or postnatal (9 studies), and the gut microbiome composition [88]. Several limitations were identified, notably the high heterogeneity between studies in terms of ELS stressors as well as in microbiome sample collection and analyses (Box 2). On the contrary, several commonalities between studies were also identified, with only 2 of the studies not finding any association. Mostly, despite some extent of conflicting results, ELS was associated with lower *Bifidobacterium* species and higher levels of Proteobacteria (typically Enterobacteriaceae) in the newborns exposed to ELS. No studies were found using psychobiotics (interventional) and few were monitoring for probiotics use. In fact, the included studies generally lack in monitoring important confounding factors such as diet and antibiotic use.

Box 2 **Limitations of human studies on ELS and microbiome composition [41].**

- High heterogeneity in the ELS stressors studied, with some stressors assumed (i.e., effect of long-term institutionalized care on microbiome in children) but not confirmed (i.e., no emotional state measure reported).
- Few studies used adult biomarkers of stress to complement/confirm self-reported or interview-reported ELS.
- Heterogeneity in the collection, processing, and analyses of stool samples.
- The impact of medications is difficult to estimate (e.g., antiretroviral therapy, antidepressants, antibiotics, etc.).
- Diet not monitored.
- Participants age range very large (from newborns to adults with PTSD ^‡^)

**^‡^ based on the retrospective history of ELS exposure.**

While behavioral assessments on their own provide limited mechanistic information in humans, identifying specific biological markers such as inflammatory and other circulating molecules, beyond the ones we already have, to use as proxy for clinical trials is a must to gather a more comprehensive picture [89]. A number of psychobiotics have shown their potential in positively impacting ELS-induced consequences. Liu et al. found that the administration of a *Lactiplantibacillus plantarum* strain in mice subjected to maternal separation significantly reduced inflammation while increasing levels of serotonin in multiple areas of the brain [90]. Another group has also shown that supplementation with a probiotic formulation containing *Lactobacillus helveticus* and *Lacticaseibacillus rhamnosus* (Lacidofil®) alleviated the deleterious impacts of ELS on the fear retention and extinction trajectory and neuronal activation in the brain [91]. Another study found that administering this same probiotic formulation to rats subjected to chronic unpredictable stress reduced microglia immunoreactivity, suggesting both a neuroprotective effect of the psychobiotic as well as a reduction of neuroinflammatory pathways associated with microglia activation [92]. 

Under chronic stress, the microglia remains in a constant state of activation, which has been associated with increased production of inflammatory cytokines, creating a hostile environment that promotes neuronal damage. Microglia activation can come from pathogen-associated molecular patterns (PAMPs) or damage-associated molecular patterns (DAMPs) [93]. These can become more prevalent in the brain in a state of chronic stress or in certain NDDs but can also reach the brain from other sources. In the gut, stress can affect the integrity of epithelial tight junctions [7], in addition to impairing the differentiation intestinal stem cells into protective cells by stimulating indole-3-acetic acid (IAA) production by some *Lactobacilli* strains [94]. These effects increase gut permeability which in turn causes more microbial products to reach the bloodstream. It is believed that some of those bacterial products that should not normally reach systemic circulation may accentuate stress-induced microglial activation and exacerbate neuroinflammation. Psychobiotics could counteract these effects by acting on both the intestinal barrier and by secreting molecules that positively regulate brain function and reduce inflammation.

## 5. The Immune System and NDDs

Recent research emphasizes the essential role of inflammation and immune system dysregulation in the pathogenesis of common NDDs, including AD, frontotemporal dementia (FTD), ALS, and PD. Evidence suggests that early activation of innate immune pathways, mostly by hallmarks of NDDs such as misfolded proteins or aggregated substances, could be an early cause rather that a consequence of neurodegeneration. This is supported by findings that reported the correlation between severe infections and accelerated cognitive decline in AD, linked to increased levels of peripheral tumor necrosis factor alpha (TNF-α) and the beneficial role of non-steroidal anti-inflammatory drugs in lowering the disease risk. Furthermore, genetic analyses have identified specific genes associated with innate immune pathways and microglial cells, suggesting a pathogenic role for neuroinflammation in AD. These include genes coding for complement receptor 1 (CR1), myeloid cell-expressed membrane-spanning 4-domains subfamily A member 4E (MS4A4E), and CD33, which is involved in suppressing pro-inflammatory cytokines and amyloid-β clearance by microglial cells. FTD, the second most common dementia type after AD, also involves neuroinflammation, evidenced by elevated TNF-α and Transforming growth factor beta (TGF-β) levels in cerebrospinal fluid and increased microglial activation. FTD is linked to mutations in the *GRN* gene that results in reduced levels of progranulin. Progranulin deficiency leads to an imbalanced inflammatory response, suggesting that neuroinflammation isn’t merely a secondary effect but integral to disease pathogenesis [95]. PD is the second most common NDD overall after AD [96]. Activated microglia play a key role in the progression of PD by contributing to neuroinflammation. Research has shown that in PD brains, microglia are abnormally activated, resulting in high levels of HLA-DR expression in affected brain areas. These HLA-DR molecules facilitate the presentation of antigens to CD4+ T lymphocytes. This process, along with the secretion of inflammatory mediators, leads to the degeneration of dopaminergic neurons [97]. In line with these reports, studies on ALS/FTD pathologies highlighted the early and prominent role of microglia and astrocyte activation in pathogenesis. Key observations come from patient autopsies, showing characteristic neuronal inclusions and cell loss alongside glial activation. Human imaging studies and animal model research further confirm that neuroinflammation occurs early in the disease process, with microglial activation closely tied to disease progression [98].

It is becoming increasingly evident that the gut microbiota plays a pivotal role in immune regulation, inflammation, and the pathophysiology of NDDs. Emerging research elucidates how the gut microbiota directly impact immune system by facilitating interactions between bacterial molecules (e.g., LPS, peptidoglycans) and immune cells (e.g., dendritic cells, macrophages), thereby modulating immune responses [99]; and indirectly, by producing various metabolites such as polyamines and SCFAs [100]. Such biomolecules modulate immune responses, both locally within the gut and systemically, by influencing the proliferation and function of Treg cells, as well as the production of anti-inflammatory cytokines. This modulation is crucial for maintaining immune homeostasis and preventing overactive immune responses that can lead to chronic inflammation. While balanced gut microbiota plays a crucial role in controlling inflammation and maintaining a healthy gut barrier, on the contrary, dysbiosis has been linked to altered immune responses, increased intestinal permeability, and disruption of the BBB. In germ-free mice, a significant reduction of both occludin and claudin-5 (but not ZO-1) expression has been identified in the frontal cortex, hippocampus and striatum, as compared with pathogen-free mice [101]. This facilitates the entry of pro-inflammatory cytokines into the CNS, promotes neuroinflammation, and subsequently contributes to the progression of neuronal damage and degeneration [102]. In a preclinical study, the authors reported that the gut microbiota is essential for the microglia maturation and function. Indeed, germ-free mice exhibited microglia with defects that impaired innate immune responses but reintroducing a complex microbiota or SCFAs partially restored microglia functionality [103]. Another study also supported that the presence of gut microbiota is crucial for microglial activation and is necessary for the motor deficit phenotype to fully develop in mice models of synucleinopathies, such as PD. Notably, the neuroimmune response activation was partially attributed to SCFAs which induced microglial activation. Moreover, mice with α-synuclein overexpression showed increased physical impairments when colonized with microbiota from PD patients compared to those from healthy donors, suggesting human microbiome alterations could be a risk factor for PD [104].

Given the emerging evidence highlighting the role of the gut microbiota in regulating immune system and neuroimmune responses, and the link to various neurological disorders, including NDDs, it becomes increasingly intuitive to consider psychobiotics as potential modulators within this context. While studies evaluating the direct effects of psychobiotics on neuroinflammation are limited. There are some reports that tested specific strains and partially linked their beneficial effects to immune modulation. For example, administration of *Lactobacillus plantarum* PS128 has been shown to significantly reduce neuroinflammation, by preventing gliosis, and improve cognitive function in animal models of AD [105]. Another study focused on inflammation, insulin and lipid-related genes in peripheral blood mononuclear cells (PBMCs) in PD patients. It reported that a probiotic blend containing *Lactobacillus acidophilus*, *Bifidobacterium bifidum*, *L. reuteri*, and *Lactobacillus fermentum* significantly decreased the expression of pro-inflammatory genes IL-1, IL-8, and TNF-α, while increasing the expression of TGF-β, a regulatory cytokine, and PPAR-γ, associated with anti-inflammatory processes [106]. Furthermore, in another AD mice model, the administration of SLAB51 probiotic formulation resulted in an increase in *Bifidobacterium* spp., known for their anti-inflammatory properties, and a decrease in Campylobacterales, which are associated with pro-inflammatory effects. This shift in the gut microbiota composition led to reduced plasma levels of pro-inflammatory cytokines, indicating the probiotic’s potential to modulate inflammatory pathways. Additionally, treated AD mice showed increased levels of G-CSF, a cytokine involved in systemic immune response modulation [107]. Oral supplementation with *L. helveticus* R0052 and *B. longum* R0175 combination of LPS-treated rats (development of AD-associated mechanisms) significantly decreased the elevation of both circulating and hippocampal levels of proinflammatory cytokines and attenuated the decremental effect of LPS on memory through BDNF protein expression [108]. Another interesting probiotic effect on BBB permeability had been reported in germ-free mice monocolonized with a single bacterial strain, either *Clostridium tyrobutyricum*, a butyrate-producer, or with *Bacteroides thetaiotaomicron*, an acetate- and propionate-producer. Indeed, after 3 days of oral gavage, analysis performed after Evans blue perfusion demonstrated that both probiotic strains and also sodium butyrate-treatment decreased BBB permeability which was associated with an increased in tight-junction protein expression and may be also linked to an increase of histone acetylation after sodium-butyrate or *C. tyrobutyricum* treatments [101].

Despite these promising findings, the mechanisms through which psychobiotics exert their effects on the immune system and inflammation within the MGBA remain to be fully elucidated and confirmed in randomized placebo-controlled clinical trials. The complexity of microbial communities, coupled with the diversity of immune responses and neural effects, presents a challenge in delineating causal relationships. This challenge is even more important when one considers the comorbidities that are related to dysregulated or low-grade, chronic inflammation such as the metabolic syndrome, obesity and diabetes.

## 6. Eating Behaviors, Metabolic Health, and NDDs

The multidirectional interactions existing between the brain and the GI tract suggest that diet might impact mental health and vice versa, and that dietary patterns might be altered in individuals suffering from neurological disorders. It is now widely recognized that both the CNS and ENS play a significant role in regulating food intake [109]. Food itself plays a major role in regulating appetite. Some nutrients, but also probiotics and prebiotics, might interact with the sensor neurons present in the GI tract, modulating the feeling of hunger (or satiety) and inflammatory processes. These nutrients or dietary supplements may also modulate the composition and functions of the intestinal microbiome, which in turn impact the production of metabolites of interest. For example, ingesting polyphenols modifies the microbiota, but the microbiota also enhances the effects of polyphenols and modifies them by producing metabolites that may improve the prognosis of NDDs [110,111].

Regulation of eating behaviors is complex and results from the modulation of both intrinsic factors, such as genetics, hormones, neural signals, and extrinsic factors, including environment. This regulation is even more complex in humans than other mammals due to hedonic food or social contexts. Moreover, it is important to consider that individuals are not always rational regarding food consumption. Some people act as emotional eaters, consuming food in response to a (positive or) negative stimulus, including stress, rather than a response to a physiological need. Ultimately, if it becomes a habit, this behavior might lead to the development of pathological issues such as overeating episodes or binge-eating disorders (BEDs), for instance [112]. A recent stratification of the Food4Gut cohort (ancillary study) revealed that individuals with obesity suffering from BED had slight but significant differences in gut microbiota composition and metabolomic profile compared to individuals with obesity but without BED [113].

In the context of obesity, it has been reported that proinflammatory molecules produced by adipose tissue expansion could reach the hypothalamus from the vagus nerve, thus promoting the production of neural proinflammatory mediators by the activation of endothelial and glial cells [114]. Several studies reported that a low-grade proinflammatory status reported in obesity and its related metabolic disorders have the potential to affect the brain negatively, increasing local inflammation, and altering plasticity or brain structure [115]. In patients with type 2 diabetes, insulin resistance has also been reported in the hippocampus, which is associated with alterations of learning and memory capacities [116]. In the aging population, individuals with obesity or diabetes are at high risk of AD. Another interesting mechanism in this vast network of molecules linking energy metabolism and mental health is the dual role of ghrelin. Ghrelin - a well-documented orexigenic hormone - induces, among others, hunger feelings and energy intake through the stimulation of orexigenic neurons of the central nervous system. Growing evidence also suggests a key role of ghrelin in improving neuroplasticity, neuroprotection and cognitive functions, particularly in AD and PD, potentially through the indirect inhibition of microglia activation [117,118]. Further experiments remain necessary to demonstrate these ghrelin-signaling-dependent pathways [117].

Moreover, depression reflects a negative emotion but can be subdivided in atypical depression, mainly characterized by an increase of appetite and highly palatable food consumption, and melancholic depression, typically characterized by appetite loss and decrease of body weight. In MDDs, the “loss of pleasure” or anhedonia is an intriguing symptom associated with reward-associated disorders [119]. In depression pathophysiology, several signaling pathways and molecular alterations hypotheses were investigated, including the decrease of monoamines brain levels or GABAergic neurons, for instance. Recent studies also highlighted a potential role of the endocannabinoid (eCB) system in the regulation of a plethora of metabolic and NDDs since cannabinoid receptors (i.e. CB1 and CB2 subtypes) are widely expressed in the body [119]. The endocannabinoidome has been described as a large family of lipid mediators produced from ubiquitous lipid precursors and involved in metabolism, inflammation, and behavior and thus in eating disorders as explained more recently [120]. Growing evidence also demonstrated that the neuroprotective, anti-inflammatory and antioxidant properties of the Mediterranean diet could be partly explained by beneficial modulations of the eCB system [121]. MIND diet, meaning Mediterranean-DASH Intervention for Neurodegenerative Delay, has been proposed to specifically focus on brain health and thus, to reduce dementia and cognitive decline occurring as people get older. Clinical trials are under investigation to demonstrate its efficiency and some studies have already reported improvement of cognitive function [122,123,124].

These examples demonstrate that mental and metabolic health are interconnected, and some regulatory mechanisms share similar origins, targets and signaling pathways. Eating behavior alterations could be both the cause and the consequence depending on the origin of the pathology. Many preclinical experiments carried on in rodents have reported that drug or dietary treatments impacting either mental or metabolic health, also have significant effects on the other. However, clinical trials considering both aspects are still underrepresented in the literature, especially in mild mental disorders and neurodegenerative pathologies. These missing connections between mental and metabolic health in humans should be evaluated in future clinical trials by involving experts from both health segments from the start of the project. It is of utmost interest to demonstrate that the use of treatment to prevent or decrease symptoms of cognitive decline and NDDs could also have a beneficial effect on body composition, improvement of food quality and quantity intake and possibly on the regulation of the proinflammatory status of the adipose tissue.

Although available evidence shows that probiotics can modulate the immune system and exert anti-inflammatory effects, alter the gut microbiota, produce neuroactive substances, and influence gut barrier function, these mechanisms are not well-defined at the molecular level and likely vary across different probiotic strains and individual hosts. Indeed, mechanisms involving specific metabolic or cellular pathways should be explored. Finally, the matrix or environmental factors to which the strain is exposed during production could significantly affect the end outcomes if the mechanisms were proven to depend on the presence of specific substrates for a given metabolic reaction to occur [125]. Consistent quality in probiotic sourcing for clinical trials and afterwards should be considered early and included among the key factors during the development of clinical trial protocols to limit downstream changes in therapeutic formulations. To achieve personalized clinical trials, future research should first focus on conducting studies with more rigorous designs, and well-documented and diversified outcome measures, with a greater emphasis on unraveling the mechanisms of action. This would help bridge the gap between the preventive and therapeutic applications of psychobiotics to finally unravel their full potential in managing mental and neurological disorders. But how do we move away from traditional study designs towards more innovative designs that could allow to prove causality?

## 7. Psychobiotics as Prevention versus Treatment

There is a clear dichotomy between the use of probiotics as therapeutic agents versus preventive or adjunctive agents in the context of mental and neuropsychiatric disorders. Traditionally, probiotics have been studied in a “clinical” context or disease situation, where animal models modified to express a certain pathological phenotype are administered probiotics. In humans, probiotics are primarily perceived as a supportive food supplement rather than treatment, with the main goal of improving general wellbeing, quality of life and preventing disease occurrence or progression. A recent systematic review and meta-analysis reported that out of 54 clinical trials in humans, only 13 studies (24.1%) recruited participants with diagnosed psychiatric disorders according to the Diagnostic and Statistical Manual of Mental Disorders (DSM), such as MDD, and schizophrenia, whereas 41 studies presented data of healthy participants with no diagnosed psychiatric disorder [126]. The discrepancies between studies conducted in healthy or stressed versus medically diagnosed individuals leave a gap in our understanding and exploitation of probiotics at their full potential. The inconsistency in available data may also be attributed to the high variability in trial designs, including significant variations in used strains, doses, timelines, outcome measures taken and clinical assessment tools [81]. Other challenges that can influence the effectiveness of psychobiotics as live biotherapeutic products (LBPs) include timing of administration (with respect to age or disease onset), regional diet differences, and for some strains, the matrix in which they are delivered. 

Some psychobiotics have demonstrated anxiolytic and antidepressant effects in both preclinical and clinical studies, while other studies suggested that probiotics could enhance the efficacy of conventional drugs. A recent study showed that in adults diagnosed with MDD and with an incomplete response to prescription antidepressants a supplementation with a 14-strain blend probiotic resulted in a greater improvement compared to those on placebo [127]. Given the expanding horizon of probiotic research, it is crucial to distinguish probiotics used as food supplements from the LBPs aimed to be used in patients [125,128]. As evidence grows supporting the role of probiotics in treating, not just preventing, specific CNS diseases, the conversation around developing bacteria as medicines rather than preventive measures gains even more relevance. Furthermore, interindividual variability in gut microbiota composition underscores the benefits of developing panels or cocktails of probiotic strains tailored to individual or subgroups’ needs, a concept that aligns with the principles of personalized medicine or stratified approaches [129]. The list of key variables to take into consideration while designing clinical trials is long, and it is becoming clearer that a ‘one size fits all’ approach may not be sufficient in the context of psychobiotics as LBPs.

## 8. Towards Mechanisms of Action: New Technologies for Preclinical and Clinical Investigations

A main challenge in the study of psychobiotics as biotherapeutics is the lack of a clear understanding of their full mechanisms of action. This gap in knowledge not only impose limitations on current research but also creates resistance toward the use of probiotics by patients and health care providers. This is particularly relevant in the context of mental and neurological disorders such as NDD. To this day, most NDDs are lifelong with no curative treatment. Thus, patients being presented with psychobiotics as a potential management modality would expect to at least know the primary mechanism through which the psychobiotics are acting. In addition, awareness of the detailed mechanisms of action will greatly improve our understanding of probiotic-host interactions, which will subsequently improve strain selection for future research and help tailor our supplementation to specific disease phenotypes. Key mechanisms could involve metabolites such as SCFAs and neurotransmitters as already proposed, but it is highly possible that other factors are involved. As we eliminate certain possibilities, we will move closer to understanding how psychobiotics function. To achieve this, a systematic approach is needed, and although probiotics and psychobiotics may work through multiple mechanisms biologically, which is a fascinating possibility, it is beneficial for both the industry and clinicians to identify a primary mechanism. Similar to drugs, a useful starting point would be to develop a comprehensive strain collection that is suitable for high-throughput screening (HTS) through fast, automated, or AI-assisted processes [130,131]. This can be coupled with genomics, especially whole genome sequencing and genetic modification techniques. Such approaches could help pinpoint key genes involved in certain probiotic effects and potentially facilitate the validation of probiotic activity, and even lead to the discovery of new mechanisms and therapeutic interventions.

Utilizing emerging monitoring technologies could also provide valuable insights in this pursuit. Smart devices, like watches, urinary metabolite monitors, stool sampling and consistency monitoring devices, and sleep quality and respiratory activity monitors, offer new ways to track health parameters in healthy, at-risk, or diseased populations during observational and clinical studies [132,133,134]. These technologies, paired with tailored questionnaires and the collection of blood, saliva, and stool samples using protocols suited to metagenomic and metabolomic analyses would yield rich datasets. However, maintaining compliance can pose challenges if clinical protocols become overly complex. Therefore, balancing thoroughness and simplicity is key.

## 9. Conclusions

In this review, we have embarked on an exploratory journey through the multifaceted domain of psychobiotics and the MGBA, uncovering their prominent roles across various contexts. It is evident that the study of psychobiotics has come a long way already, but the road ahead is still winding; we need to reshape our ways to study the applications of probiotics in human health. While some psychobiotic formulations like Cerebiome® are well-established and supported by several trials in participants with depression [81], it is important to remember that not all probiotics are psychobiotics. Overall, our understanding of how communities of commensal microorganisms function and interact with their hosts is improving all the time. This is largely due to the development of investigation technologies, which are increasingly precise and accessible, combined with ever greater data analysis and integration capabilities. Advances in our knowledge are leading to a better understanding of the activity of well-known probiotic strains, but also to the development of new strains that target specific mechanisms of action that have recently been highlighted. Among the many possible applications, MGBA-based interventions could help to prevent and/or treat a multitude of conditions, the prevalence of which is constantly increasing throughout the world. However, these subjects remain extremely complex and difficult to understand in their entirety: the activity of the strains, interactions with the host, production on an industrial scale, etc. all require specific specialist knowledge.

As we look into the future, we must implement novel approaches to deepen our understanding of psychobiotics, fill existing knowledge gaps, and extend their role beyond current applications. 

High-throughput sequencing and metabolomics offers a promising opportunity to personalize psychobiotic treatments. For instance, utilizing these technologies to analyze individual microbiome compositions could guide health care providers to prescribe specific probiotic strains that have been shown to improve symptoms of depression or anxiety in individuals with similar microbiome profiles. This approach mimics the precision medicine model, in which treatments are tailored to the unique genetic, environmental, and lifestyle factors of each individual. Not only will this offer a novel management modality but may be leveraged toward preventive measures in high-risk individuals.

Additionally, the intersection between artificial intelligence (AI) and microbiome research holds the potential to transform psychobiotic discovery and functional analysis. AI algorithms, trained on vast datasets of microbiome sequences and clinical outcomes, could predict the therapeutic potential of novel psychobiotic strains with high accuracy. Such technologies could also uncover complex microbial interactions within the GBA, shedding light on the underlying mechanisms of action. These predictions and our current knowledge must be supported by interdisciplinary clinical trials that entails a collaborative approach, uniting disciplines such as neuroscience, microbiology, psychology, and bioinformatics, and take into account the high inter-individual variability, as well as specific formulations and dose. Mental health’s related diseases have been associated with pleiotropic alterations along the MGBA, including gut microbiota composition and functions, and also overall and local immunity. Such studies not only contribute to our understanding of psychobiotics’ potential but also facilitate their acceptance among healthcare professionals and facilitates psychobiotics’ integration into standard treatment protocols.

An important challenge to consider for future perspectives is the ethical and regulatory considerations, particularly concerning the privacy and security of microbiome data. Thus, the development of comprehensive international guidelines and regulatory frameworks will be vital to protect individual rights and foster innovation. Furthermore, the public health implications of psychobiotics, especially their potential to mitigate the global burden of mental health disorders and NDDs, must be emphasized. For example, the widespread implementation of psychobiotics as adjunct therapies for MDD could significantly reduce healthcare costs and improve quality of life for many worldwide.

The future of psychobiotics and the MGBA carries a significant promise and potential. As we move into this novel territory, a concerted effort from the scientific community, industry stakeholders, and regulatory bodies will undoubtedly be an essential lever for accelerating the development and rapid availability of new ‘psychobiotics’ solutions. While we have everything at hand, it is a matter of uniting the right teams, with the right tools and methods and asking the right research questions.

## Data Availability

Not applicable.
